# Severe Obstructive Sleep Apnea Disrupts Vigilance-State-Dependent Metabolism

**DOI:** 10.3390/ijms232214052

**Published:** 2022-11-14

**Authors:** Felix Schmidt, Nora Nowak, Patrick Baumgartner, Thomas Gaisl, Stefan Malesevic, Bettina Streckenbach, Noriane A. Sievi, Esther I. Schwarz, Renato Zenobi, Steven A. Brown, Malcolm Kohler

**Affiliations:** 1 Faculty of Medicine, University of Zurich, 8006 Zurich, Switzerland; 2 Department of Pulmonology, University Hospital Zurich, 8091 Zurich, Switzerland; 3 Department of Chemistry and Applied Biosciences, ETH Zurich, 8093 Zurich, Switzerland; 4 Institute of Pharmacology and Toxicology, University of Zurich, 8006 Zurich, Switzerland; 5 Zurich Centre for Integrative Human Physiology, University of Zurich, 8006 Zurich, Switzerland

**Keywords:** obstructive sleep apnea, sleep, metabolomics, breath analysis, secondary electrospray ionization, mitochondria

## Abstract

The direct pathophysiological effects of obstructive sleep apnea (OSA) have been well described. However, the systemic and metabolic consequences of OSA are less well understood. The aim of this secondary analysis was to translate recent findings in healthy subjects on vigilance-state-dependent metabolism into the context of OSA patients and answer the question of how symptomatic OSA influences metabolism and whether these changes might explain metabolic and cardiovascular consequences of OSA. Patients with suspected OSA were assigned according to their oxygen desaturation index (ODI) and Epworth Sleepiness Scale (ESS) score into symptomatic OSA and controls. Vigilance-state-dependent breath metabolites assessed by high-resolution mass spectrometry were used to test for a difference in both groups. In total, 44 patients were eligible, of whom 18 (40.9%) were assigned to the symptomatic OSA group. Symptomatic OSA patients with a median [25%, 75% quartiles] ODI of 40.5 [35.0, 58.8] events/h and an ESS of 14.0 [11.2, 15.8] showed moderate to strong evidence for differences in 18 vigilance-state-dependent breath compounds compared to controls. These identified metabolites are part of major metabolic pathways in carbohydrate, amino acid, and lipid metabolism. Thus, beyond hypoxia per se, we hypothesize that disturbed sleep in OSA patients persists as disturbed sleep-dependent metabolite levels during daytime.

## 1. Introduction

The term vigilance-state describes two phases during the day, which can be sleep or wakefulness. The current understanding of sleep includes four main different sleep stages: rapid eye movement (REM) sleep (stage R) and non-REM sleep stages 1–3 (NR 1–3), whereby NR3 represents slow-wave sleep [1].

The timing and structure of sleep are determined by the interaction of a homeostatic and a circadian process [2]. The circadian rhythm is a continuous change in physical, mental and behavioral processes, which follows a 24 h cycle with the two vigilance-states wakefulness and sleep. This natural process responds primarily to light/dark and ensures body homeostasis. The master clock in the human brain coordinates a complex interplay between a myriad of biochemical processes and, thus, the human metabolome. Different metabolic reactions build a logical cascade and result in a healthy homeostasis. Vigilance-states are highly regulated [3,4] and affect metabolism [5]. Nowak et al. [5] showed via breath analysis that sleep-regulated metabolic pathways show logical patterns across different sleep stages in healthy people. These findings suggest that disturbed sleep may also affect metabolism and its regulation during sleep.

Research on the association of sleep and metabolism as well as the role of sleep disorders in the development of metabolic disorders (e.g., obesity and disturbed glucose tolerance) is a topic of interest, particularly in patients with OSA, one of the most prevalent disorders that disturbs sleep. Recurrent pharyngeal airway collapse results in intermittent hypoxemia and arousals that fragment sleep considerably, leading to increased sympathetic activity and autonomic disturbance [6]. Furthermore, intermittent hypoxemia leads to oxidative stress, which can cause molecular damage and can thereby exacerbate chronic diseases [7,8]. Finally, impaired sleep is also associated with major physiological and psychological sequelae, such as impaired glucose and lipid metabolism, development of cardiovascular diseases, and impaired psychological and social functioning, with enormous socioeconomic consequences [9]. For these reasons, OSA can be interpreted as a systemic disease and—if left untreated—can have a variety of systemic and metabolic consequences.

Different techniques from cell cultures over animal models and blood and tissue examinations in humans were used to study metabolic changes in OSA and enhance the understanding of altered metabolic pathways in OSA. Most studies used only models of intermittent hypoxia or invasive and usually non-continuous investigations of, e.g., blood or tissue samples [10,11,12]. Breath analysis offers a noninvasive, fast and patient-friendly method to obtain metabolic insights into the human body and allows repetitive measurements. This noninvasive approach allows breath sampling during sleep or during clinical routine and reveals the lung aerosol levels of thousands of metabolites with a single breath. Our group has recently shown, by using exhaled breath analysis during sleep, that the levels of hundreds of metabolites are vigilance- and sleep-stage dependent in healthy humans [5]. Additionally, breath analysis studies indicate that OSA patients vs. controls show an altered breath pattern [13]. These two findings raise the question of whether these altered breath patterns in OSA patients are related to hypoxia per se, or also possibly to disrupted vigilance-state-dependent metabolism due to disrupted sleep. Thus, the objective of the secondary analyses was to quantify vigilance- and sleep-stage-dependent metabolic pathways in OSA patients, which were previously identified in healthy subjects, as well as to determine whether differences in vigilance- and sleep-stage-dependent metabolic pathways are detectable in morning breath analysis between symptomatic OSA patients and non-sleepy people without relevant OSA (controls) and to translate these findings into the context of systemic pathophysiological mechanisms in OSA disease.

## 2. Results

### 2.1. Overall Results and Demographics

**Figure 1:**
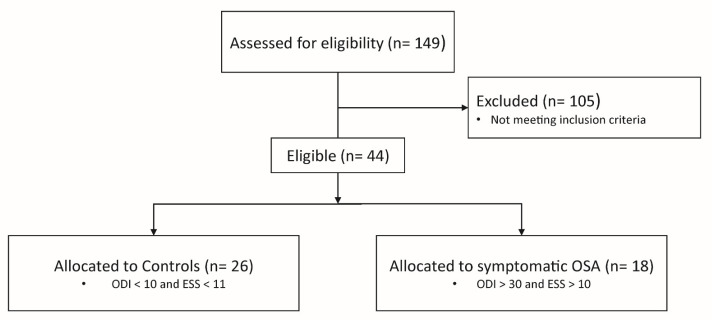
Study Flowchart.

Of 149 patients screened, 44 patients were eligible and included either into the symptomatic OSA or the control group (Figure 1).

In total, 26 patients referred to the sleep clinic were suitable as controls (no relevant OSA with ODI < 10/h and ESS < 11). This group showed a median [25%, 75% quartiles] ODI of 5.0 [3.0, 7.0] events/h and a median [25%, 75% quartiles] ESS of 6.0 [4.2, 7.8] points. A total of 18 patients with severe OSA (ODI > 30/h) and daytime sleepiness (ESS > 10) were characterized by a median [25%, 75% quartiles] ODI of 40.5 [35.0, 58.8] events/h and a median [25%, 75% quartiles] ESS of 14.0 [11.2, 15.8] points. Table 1 shows baseline characteristics. Groups were well balanced except for a relevant difference in BMI and a slight difference in selected comorbidities.

### 2.2. Sleep-Stage-Dependent and OSA-Dependent Breath Metabolites

**Figure 2:**
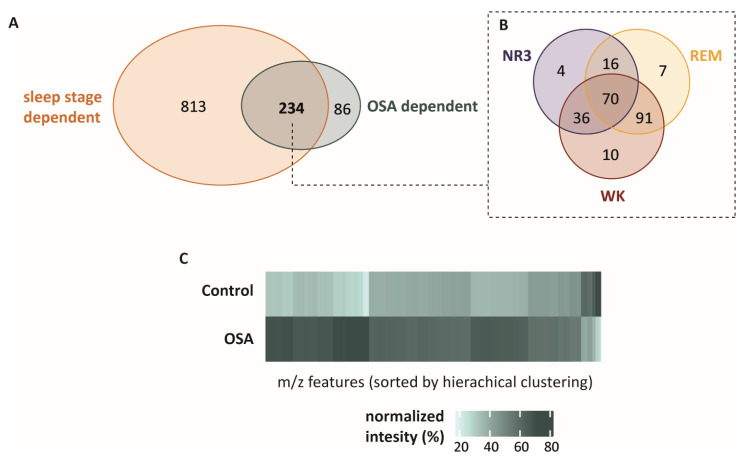
(**A**) Venn-diagram showing 1133 breath features that are either sleep-stage- or OSA-dependent. (**B**) A total of 234 sleep-stage-dependent features were also dysregulated in OSA patients compared to controls (*p* ≤ 0.05). Furthermore, these overlapping breath features are associated with different sleep phases (NR3 = non-rapid-eye-movement sleep—stage 3; REM = rapid-eye-movement sleep; WK = wakefulness). (**C**) The heat map shows the differences between the medium intensity of breath features in the symptomatic OSA group and the control group for these 234 m/z features. OSA patients show higher intensities (dark green) in most of the breath features. The intensities are represented as % of the sum of both groups.

Findings from previous studies [5,13] enable us to consider 1047 breath features as sleep-stage-dependent and 320 as OSA-dependent (Figure 2A). The intersection in the Venn diagram (Figure 2B) shows that 234 breath features are sleep-stage-dependent and OSA-dependent. In the next step, we focused on these 234 breath features that are sleep-stage-dependent and OSA-dependent and compared them between the control and symptomatic OSA group. The majority of OSA- and sleep-stage-dependent breath features showed higher intensities in the symptomatic OSA group compared to controls (Figure 2C).

### 2.3. Differences in Major Metabolic Pathways Related to Sleep Architecture between Symptomatic OSA and Controls

**Figure 3:**
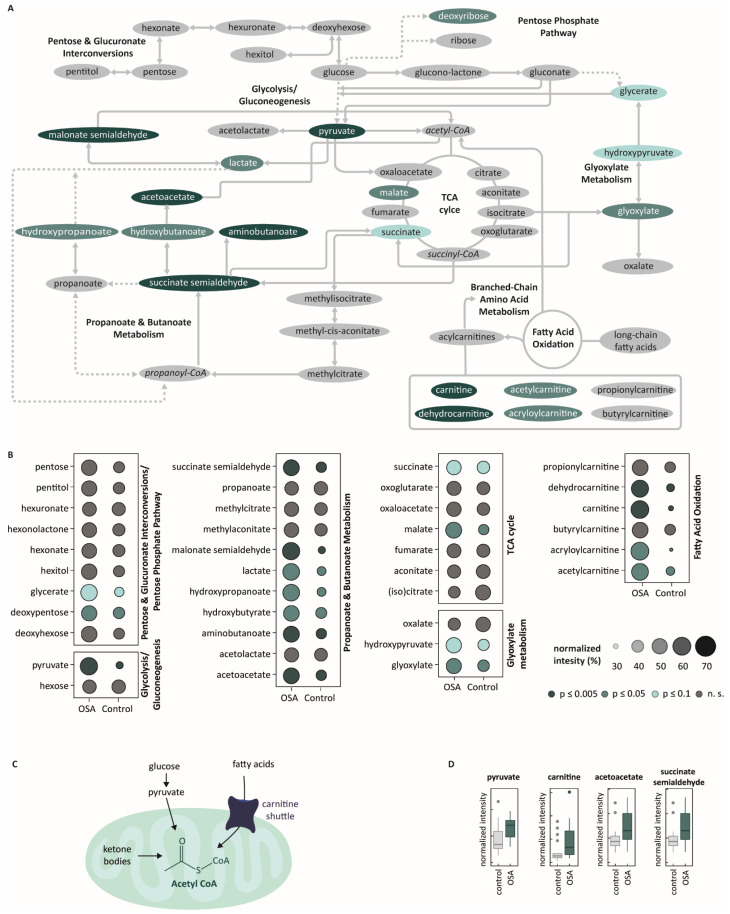
Sleep-stage-dependent metabolites in symptomatic OSA patients compared to controls. (**A**) Map of major metabolic pathways related to sleep architecture. All compounds on this map are vigilance-state-related (1). Metabolites that are elevated in OSA patients compared to controls are highlighted in green (dark green: *p* ≤ 0.005; green: *p* ≤ 0.05; light green: *p* ≤ 0.1). (**B**) Intensities of identified sleep-stage-dependent metabolites of major pathways in symptomatic OSA patients vs. control. Dot size represents intensity in %; colors indicate the level of significance of the difference between the two groups (n.s.: not significant). (**C**) Schematic and hypothetical mitochondrial burnout due to sleep–wake disturbance in OSA. (**D**) Boxplots of elevated mitochondrial-metabolism-associated precursors in breath of OSA patients during daytime. Boxplots (center line: median; box limits: 25th and 75th percent quantile; whisker length: 1.5 interquartile range).

These 234 metabolites include 38 representatives of major biochemical pathways that have been identified in [5] with MS-MS spectra or exact mass and pathway mapping as showing significant and characteristic changes across different vigilance states. A total of 18 out of these 38 compounds also showed evidence for a difference in the two groups (OSA vs. controls). For all details, please see Table S2. Figure 3A gives an overview of the identified metabolites in the context of related biochemical pathways. The daytime breath profile of symptomatic OSA patients indicates differences in lipid metabolism compared to controls. Symptomatic OSA patients showed moderate to strong evidence for higher levels of long-chain fatty acid transport carnitine derivatives. Furthermore, we observed differences in catabolic metabolic conditions. Ketone bodies such as acetoacetate showed strong evidence and hydroxybenzoate showed moderate evidence to occur in higher concentrations in symptomatic OSA patients. Additionally, we observed variations between the two groups in the amino acid metabolism. Specifically, compounds such as amino butanoate, malonate semialdehyde, and succinate semialdehyde involved in glutamate and GABA metabolism as well as in multiple other amino acid pathways showed strong evidence for a higher occurrence in symptomatic OSA patients. Moreover, these compounds are also part of the butanoate and/or propanoate pathway. Figure 3B presents various compounds from the butanoate and/or propanoate pathway, which showed no or weak evidence for a difference between the two groups. Further differences in the carbohydrate metabolism were detected: The pentose cycle seemed to be affected, but strikingly, main metabolites from the glycolysis, gluconeogenesis, and glyoxylate cycles differed between both groups. Glycerate and lactate compounds from the pyruvate turnover showed moderate evidence and pyruvate itself was found with strong evidence for higher concentration in the symptomatic OSA group. Substrates such as malate and succinate from the tricarboxylic acid cycle (TCA) as well as glyoxylate and hydroxypyruvate out of the glyoxylate cycle were elevated in symptomatic OSA patients. Boxplots of named compounds can be found in Figure S1. Strikingly, most of the identified metabolites that showed evidence for differences between the groups could be assigned to central energy processes, and in particular, to sources of mitochondrial fuel (pyruvate, carnitine and ketone body components).

## 3. Discussion

This comparison of exhaled breath during wakefulness in symptomatic OSA vs. controls provides evidence for a difference in vigilance-state-dependent metabolism between these groups, particularly in major axes of cellular metabolism such as the lipid, amino acid, and carbohydrate pathways. Additionally, the findings suggest that metabolites associated with acetyl-CoA and mitochondrial activity remain altered in symptomatic OSA patients during daytime. The direct pathophysiological effects of obstructive apneas are well understood. However, limited knowledge exists about the systemic and metabolic consequences of OSA, and some of these findings are contradictory [14]. These translational results from breath analysis may help to understand systemic changes in OSA on a molecular level.

### 3.1. Sleep–Wake-State-Dependent Metabolism

Nowak et al. [5] showed that the activity of most major axes of cellular metabolism and TCA cycle activity are strongly sleep–wake-state-dependent in healthy people. Notably, the pathways found to be upregulated in symptomatic OSA mostly correspond to those downregulated in slow-wave sleep (NR 3) of healthy subjects. Interestingly, upregulated parts of propanoate and butanoate metabolism in symptomatic OSA patients were observed during the day, and the same metabolic features were upregulated in the REM phase during sleep in a small healthy cohort [5]. These findings suggest that the arousal-induced sleep fragmentation in OSA patients may lead to insufficient metabolic activity during specific sleep phases, and these differences persist in symptomatic OSA patients metabolically even during daytime. In addition, accumulation of aldehydes and other compounds related to butanoate/propanoate metabolism indicates that higher lipid peroxidation occurs in OSA patients and, presumably, increased oxidative stress. In healthy subjects, levels of these metabolites are decreased specifically during the deep sleep stage of NR sleep (NR3) [5], which would be disrupted in OSA patients.

### 3.2. Lipid Metabolism of Symptomatic OSA

Reviewing our findings concerning lipid metabolism, we suggest that the higher levels of carnitine derivatives observed in OSA patients indicate modifications in terms of degradation/transport of fatty acids and storage of acetyl groups between symptomatic OSA and controls. Fatty acids are transported to the mitochondria and processed there via beta-oxidation. Functional changes in mitochondria have impact on energy metabolism and are associated with cellular damage and loss of energy [15,16]. Kim et al. [16] showed that mitochondrial DNA (mtDNA) copy number was reduced in patients with OSA, suggesting mitochondrial dysfunction in the genomic DNA of whole-blood cells. Further, they showed an association between the severity of OSA and decreased mtDNA copy number, suggesting more oxidative stress in severe OSA. It is known that hypoxia leads to increased reactive oxygen species (ROS) [17]. On the other hand, carnitine, a natural compound in the fatty acid oxidation pathways, has an antioxidant effect and decreases lipid peroxidation [18]. Increased carnitine and carnitine derivates could be a possible mechanism of the body to reduce oxidative stress in OSA, or simply an indication of reduced mitochondrial function to process them. Different studies in human cells could show that carnitine reduces oxidative stress and, therefore, reduces cardiovascular risk [19,20]. Furthermore, beta-oxidation is considered the most efficient way of the body to produce ATP. Because oxidation of fatty acid requires a high oxygen supply, impaired oxygen supply could lead to reduced beta-oxidation [21].

OSA is a risk factor for metabolic disorders [22]. How OSA affects metabolism is complex and different pathways and mechanisms have been discussed in the literature. For example, McNicholas et al. [23] discussed several ways how OSA may influence metabolism. Inflammation and oxidative stress, neurohumoral changes, glucose homeostasis, and sympathetic nervous system activation may play a role [23]. Several studies assessed the association of lipid metabolism and OSA. A study showed that OSA could lead to a worsening of the lipid metabolism in non-obese patients [24]. Other studies in OSA even differentiated between REM and NR regarding the lipid profile [25]. Not only did Xu et al. [25] show that AHI_REM_ was independently associated with increased levels of triglyceride (TG), but they also provided an independent association of AHI_NREM_ with increased levels of LDL. This suggests that both REM and NR sleep seem to have an impact on lipid metabolism [25]. Additionally, observed dysregulation in GABA and glutamate metabolism supports recent findings in the OSA literature [26,27].

### 3.3. Carbohydrate Metabolism of Symptomatic OSA

Metabolites (i.e., deoxyribose, glycerate, pyruvate, and lactate) involved in the carbohydrate metabolism showed evidence to be higher in patients with symptomatic OSA when compared to the control group. This finding may be partially related to the higher BMI in our OSA group, but higher BMI alone does not give conclusive information about metabolic health. Research on metabolically healthy obese subjects and metabolically unhealthy obese subjects (MUO) suggests that for a certain BMI, the risk of cardiometabolic disease and death varies substantially among subjects [28]. However, interventional studies also suggested an altered hypothalamus–pituitary–adrenal axis in patients with sleep apnea [29]. This disruption results in generally higher norepinephrine and cortisol levels when OSA is not treated, which, in turn, stimulates glycogenolysis [30]. Furthermore, patients with OSA have an increased prevalence of insulin resistance as well as type 2 diabetes, which is also in line with an increased carbohydrate metabolism among this population [31]. Real-time breath analysis may identify especially vulnerable subgroups among patients with OSA, where the carbohydrate metabolism is disproportionately altered.

### 3.4. Mitochondrial Burnout in OSA

Our results emphasize that most of the identified metabolites have a strong association with processes “feeding” acetyl-CoA-dependent mitochondrial pathways (Figure 3C,D). This opens up many novel research questions and, furthermore, generates new hypotheses. A new hypothesis this work puts forth is that symptomatic OSA patients may suffer from a “burnout” of mitochondria. We observed that symptomatic OSA patients have a higher amount of mitochondrial-metabolism-associated precursors in their breath. A conclusion could be that these patients have a fundamental change in oxidative-phosphorylation-driven metabolic turnover that persists into daytime. Consistent with this idea, symptomatic OSA patients show sleepiness and poor concentration over the day and suffer from a lack of energy. One could compare this phenomenon with the burnout of beta cells of Langerhans island cells in the pancreas of diabetes patients [32] where insulin resistance increasingly leads to the dysfunction or dying of beta cells. The same can possibly occur within mitochondria in symptomatic OSA patients. Hypoxia [33] and the described vigilance-state-dependent metabolic distortion can lead to a mitochondrial burnout. The mitochondrial energy metabolism processes become insufficient, inefficient, and finally pathological. The influential effect of mitochondrial burnout was recently described in other relevant areas [34,35].

### 3.5. Limitations

This secondary analysis contained inherent limitations. We identified signals of predefined vigilance-state-dependent metabolic distortion in symptomatic OSA patients during daytime. However, the results need to be further validated in a prospective longitudinal day and whole-night breath analysis in symptomatic OSA patients to cover all vigilance-state-dependent pathways. Secondly, we were able to identify 38 compounds among 234 sleep- and OSA-dependent molecules. However, a large fraction of the 234 compounds remains unidentified or ambiguously identified due to the complexity of breath feature identification from mass spectrometry. Thus, results might be affected by a selection bias. An identification of more compounds would give a broader overview of up- or down-regulated metabolic pathways and focus areas, further confirming our hypotheses. Finally, this secondary analysis did not allow matching according sex or BMI. Consistent with the disease that we study, a good balance according to BMI was not possible in this secondary analysis, because of the known relationship between OSA and BMI. Thus, an impact of obesity on metabolic effects in the systematic OSA group is possible, setting the stage for a “chicken and egg” phenomenon in which negative metabolic trends might reinforce one another.

## 4. Materials and Methods

### 4.1. Study Design and Setting

This secondary analysis used data from a prospective longitudinal cohort study by Nowak et al. [5] and a prospective, cross-sectional nested case–control study by Nowak et al. [13]. These findings of vigilance-state-dependent metabolites in healthy subjects [5] were further investigated in OSA patients and controls to assess alterations in those metabolites in a clinically relevant group comparison. Patients were recruited from 2016 to 2018 in the outpatient sleep clinic of the department of pulmonology at the University Hospital Zurich (USZ). A total of 149 Continuous Positive Airway Pressure (CPAP) naïve participants with clinically suspected OSA were enrolled. According to their Epworth Sleepiness Score (ESS) and oxygen desaturation index (ODI), participants were stratified into four different groups (Table S1).

For this secondary analysis, the dataset was limited to symptomatic patients with OSA and patients with neither relevant OSA nor daytime sleepiness to allow a comparison between these two groups (symptomatic OSA vs. controls with no or only mild non-symptomatic OSA). Symptomatic OSA was defined as ODI > 30 events/h and an ESS > 10 points.

The local ethical committee approved the study protocol (KEK-ZH 201600384). The experiments were conducted in accordance with the Declaration of Helsinki, principles of Good Clinical Practice, and written informed consent was obtained from all participants before participation. Results were reported according to STROBE guidelines.

### 4.2. Study Participants

Patients from the previous trial [13] with either symptomatic OSA or controls (neither relevant OSA nor daytime sleepiness) were eligible for this secondary analysis. Inclusion criteria for the group with symptomatic OSA were age between 18 and 85 years, ODI > 30/h in an in-laboratory polygraphy, and ESS > 10 points. Controls were selected according to ODI < 10/h and ESS < 11 points. No further exclusion criteria, e.g., drugs or comorbidities, were used. Patients who did not fulfill the inclusion criteria for the OSA group or the control group (n = 105) were excluded (Figure 1).

### 4.3. Variables, Data Sources, and Measurement

The main outcome of interest was a potential difference in vigilance-state-dependent metabolites in symptomatic OSA vs. controls. Apnea-Hypopnea index (AHI), ODI, and ESS were used to characterize the severity of OSA. Conventional AHI thresholds of ≥5, ≥15, and ≥30 (mild, moderate, and severe, respectively) were used. Sex, age, BMI, smoking status, lung function parameters, and comorbidities were collected to compare baseline characteristics between the two groups. With major metabolic pathways, we describe the most important human biochemical pathways such as glycolysis, Krebs’ cycle, oxidative phosphorylation, the pentose phosphate pathway, the urea cycle, fatty acid ß-oxidation, and gluconeogenesis, which build the backbone axes for lipid, amino acid, and carbohydrate metabolism.

#### 4.3.1. SESI-HRMS Breath Measurements

**Figure 4:**
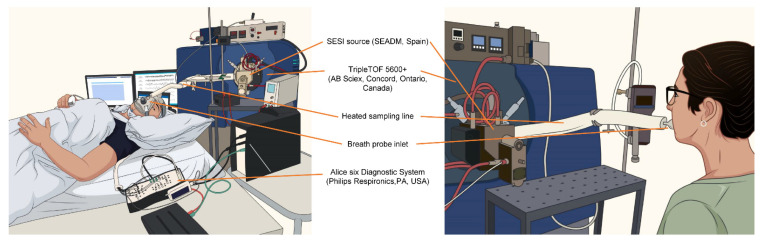
Setup of longitudinal whole-night measurements in healthy participants (**left**) and cross-sectional breath measurement during the day in OSA-suspected patients (**right**).

Datasets from longitudinal whole-night secondary electrospray ionization high-resolution mass spectrometry (SESI-HRMS) measurements [5] and cross-sectional daytime SESI-HRMS measurements [13] were analyzed. Vigilance-state-dependent breath feature matrices from Nowak et al. [5] were used to identify differences in symptomatic OSA and control subjects from Nowak et al. [13]. Exhaled breath was analyzed by SESI-HRMS using a commercial SESI source (SEADM, Boecillo, Spain) coupled to a high-resolution TripleTOF 5600+ mass spectrometer (AB Sciex, Concord, ON, Canada). Detailed information on exhalation procedures, settings of the SESI source, and the mass spectrometer are presented in the referenced publications [5,13]. For an overview, see Figure 4.

#### 4.3.2. In-Laboratory Sleep Study

In-laboratory cardiorespiratory polygraphies (RPs) were recorded by the Alice 6 Diagnostic System (Philips Respironics, Pittsburg, PA, USA), scored with validated Somnolyzer 24 × 7 software (Philips Respironics, Pittsburg, PA, USA), and reviewed manually according to the guidelines of the American Academy of Sleep Medicine [36]. The estimated ODI value was used for group stratification and AHI for descriptive baseline characteristics.

#### 4.3.3. Questionnaires

The ESS is an 8-item Likert-based questionnaire, the most commonly used measure for clinicians and researchers to measure daytime sleepiness [37]. The participant is asked to estimate the propensity to doze off in 8 different situations, thereby referring to everyday life during the previous few weeks to few months [38]. Each of these situations can be rated from 0 to 3: 0 = “would never doze”, 1 = “slight chance of dozing”, 2 = “moderate chance of dozing”, and 3 = “high chance of dozing”. The total score ranges from 0 to 24 points [39]. Total scores of ≥11 are defined as excessive daytime sleepiness [40]. We used the validated German version of the ESS for group stratification [41].

### 4.4. Bias/Study Size

To conduct a proof-of-concept analysis as relevant as possible to the clinical setting using the data from the cohort study in patients with suspected OSA, we limited the study population used in this secondary analysis to symptomatic OSA and control. The source population of this dataset is a representative sample of our target population. We, therefore, tried to reduce selection bias. To minimize information bias, we used our knowledge and experience from earlier OSA breath research experiments, which can somehow count as pilot tests and training of the team. The breath measurements were standardized and conducted by only two trained experts to avoid observer bias.

### 4.5. Data Processing and Statistical Methods

#### 4.5.1. Data Preprocessing

All mass spectral data were analyzed with MATLAB R2020a (The MathWorks, Natick, MA, USA) and R 4.0.5 (R Core Team, Auckland, New Zealand). Mass spectra obtained from exhaled breath were preprocessed with MATLAB as described in [5,13]. In short, mass spectra were interpolated, aligned, and a peak list was generated from the average breath spectrum. The peak list of identified vigilance-state-dependent breath compounds in healthy subjects [5] was used to identify differences concerning these compounds in a clinically relevant OSA and control group from the data in [13].

#### 4.5.2. Data Analysis and Statistics

To analyze the breath feature matrix, two-sided Mann–Whitney-U tests were applied for group comparisons. False discovery rates (*q*-values) were calculated using Storey’s procedure [42] to account for multiple hypothesis testing.

Baseline characteristics with normal distribution were analyzed with parametric statistics. Non-normal data were described with nonparametric statistics. Values are presented as mean (SD) or median (25%, 75% quartiles) unless otherwise stated. A two-sided *p*-value of <0.05 was considered as statistically significant.

## 5. Conclusions

Overall, the results of this secondary analysis provide plausible and confident signals for a difference in vigilance-state-dependent metabolism in symptomatic OSA patients compared to controls. This proof-of-concept work translates recent findings of sleep-regulated metabolic pathways into the large population of patients with OSA. This indicates that specific metabolic pathways are persistently up- or down-regulated during daytime wakefulness in symptomatic OSA patients, likely due to alterations during sleep and, therefore, disrupted metabolic processes at night. This noninvasive and innovative breath analysis approach enables the formulation of new hypotheses and presents new promising research targets about the metabolic consequences of OSA. Additionally, this work presents metabolic insight, which helps to explain clinical observations in terms of the systemic pathology of OSA. Furthermore, these results encourage the unravelling of the not fully understood metabolic processes with future clinical studies, which could lead to a diagnostic use of breath analysis in OSA.

## Figures and Tables

**Table 1:** Patient characteristics of the symptomatic OSA group and Control group.

	Control Group	Symptomatic OSA
N (%)	26 (59.1)	18 (40.9)
Sex = Male, N (%)	17 (65.4)	13 (72.2)
Age in years (median [25%, 75% quartiles])	46.0 [39.8, 56.2]	51.0 [43.5, 54.5]
BMI in kg/m^2^ (mean (SD))	28.0 (6.4)	33.1 (6.7)
**Smoking state**		
Non-Smoker, N (%)	11 (42.3)	7 (38.9)
Smoker, N (%)	5 (19.2)	7 (38.9)
Ex-Smoker, N (%)	10 (38.5)	4 (22.2)
**OSA parameters**		
AHI (median [25%, 75% quartiles]) in events/hour	5.0 [2.2, 6.8]	40.5 [30.5, 53.5]
ODI (median [25%, 75% quartiles]) in events/hour	5.0 [3.0, 7.0]	40.5 [35.0, 58.8]
ESS (median [25%, 75% quartiles]) in points	6.0 [4.2, 7.8]	14.0 [11.2, 15.8]
**Lung function**		
FEV1 in % predicted (mean (SD))	102.4 (15.0)	94.9 (18.5)
FVC in % predicted (mean (SD))	106.3 (16.1)	96.1 (15.0)
FEV1/FVC (mean (SD)) (%)	78.0 (5.6)	79.1 (7.9)
**Comorbidities**		
Coronary artery disease, N (%)	4 (15.4)	6 (33.3)
Hypertension, N (%)	4 (15.4)	5 (27.8)
Diabetes mellitus type 2, N (%)	2 (7.7)	2 (11.1)
Dyslipidemia, N (%)	0 (0.0)	2 (11.1)
COPD, N (%)	2 (7.7)	2 (11.1)
Asthma, N (%)	3 (11.5)	0 (0.0)
Thyroid disorder, N (%)	2 (7.7)	1 (5.6)

Definition of abbreviations: SD—standard deviation; BMI—Body mass index; AHI—apnea-hypopnea-index; ODI—oxygen desaturation index; ESS—Epworth Sleepiness Scale; FEV1—forced expiratory volume in 1 second; FVC—expiratory forced vital capacity; COPD—chronic obstructive pulmonary disease; control subjects with ODI < 10 and ESS < 11; symptomatic OSA—subjects with ODI > 30 and ESS > 10.

## Data Availability

For data availability, we would like to refer to previous published research from Nowak et al. [5,13]. The links to publicly archived resources can be found in the online article versions mentioned above. Any additional information required to reanalyze the data reported in this paper is available from the lead contact upon request.

## Supplementary Materials

The following supporting information can be downloaded at: https://www.mdpi.com/article/10.3390/ijms232214052/s1, Supplementary Table S1: Stratification of 149 suspected OSA patients out of cross-sectional nested case–control study by [13]. This secondary analysis focuses on stratification 2; Supplementary Table S2: A total of 18 identified compounds showed weak to strong evidence for a difference in breath profiles of symptomatic OSA patients compared to controls; Supplementary Figure S1: A total of 15 compounds with moderate or strong evidence for a difference are shown in boxplots; ICMJE disclosure form of all authors.
